# Data set for transcriptional response to depletion of the Shoc2 scaffolding protein

**DOI:** 10.1016/j.dib.2016.03.012

**Published:** 2016-03-09

**Authors:** Eric C. Rouchka, Myoungkun Jeoung, Eun Ryoung Jang, Jinpeng Liu, Chi Wang, Xiaohong Li, Emilia Galperin

**Affiliations:** a Department of Computer Engineering and Computer Science, University of Louisville, Louisville, KY 40292, United States; b Kentucky Biomedical Research Infrastructure Network Bioinformatics Core, University of Louisville, Louisville, KY 40292, United States; c Department of Molecular and Cellular Biochemistry, University of Kentucky, Lexington, KY 40536, United States; d Markey Cancer Center and Division of Biostatistics, University of Kentucky, Lexington, KY 40536, United States; e Department of Anatomical Sciences and Neurobiology, University of Louisville, Louisville, KY 40292, United States; f Department of Bioinformatics and Biostatistics, University of Louisville, Louisville, KY 40292, United States

## Abstract

The Suppressor of Clear, *Caenorhabditis elegans* Homolog (SHOC2) is a scaffold protein that positively modulates activity of the RAS/ERK1/2 MAP kinase signaling cascade. We set out to understand the ERK1/2 pathway transcriptional response transduced through the SHOC2 scaffolding module. This data article describes raw gene expression within triplicates of kidney fibroblast-like Cos1 cell line expressing non-targeting shRNA (Cos-NT) and triplicates of Cos1 cells depleted of SHOC2 using shRNA (Cos-LV1) upon activation of ERK1/2 pathway by the Epidermal Growth Factor Receptor (EGFR). The data referred here is available in NCBI׳s Gene Expression Omnibus (GEO), accession GEO: GSE67063 as well as NCBI׳s Sequence Read Archive (SRA), accession SRA: SRP056324. A complete analysis of the results can be found in “Shoc2-tranduced ERK1/2 motility signals – Novel insights from functional genomics”(Jeoung et al., 2016) [1].

**Specification Table**

**Table:** 

Subject area	*Biology*
More specific subject area	*Bioinformatics and cell signaling*
Type of data	*Transcriptome, table, figure*
How data was acquired	*High-throughput RNA sequencing using Illumina HiSeq 2500*
Data format	*Raw, fastq files*
Experimental factors	*RNA isolation, cDNA library construction and sequencing*
Experimental features	*Transcriptome analysis of Cos1 cells depleted of SHOC2 using:*
*Cos1 cells expressing non-targeting shRNA (Cos-NT) (control; n=3);*
*Cos1 cells expressing SHOC2 specific shRNA (Cos-LV1) (n=3)*
Data source location	*Lexington, KY, USA*
Data accessibility	*The data is available with this article and via NCBI׳s GEO through the direct link*http://www.ncbi.nlm.nih.gov/geo/query/acc.cgi?acc=GSSE67063*. GEO: GSE67063*

**Value of the data**

• While the activation of RAF, MEK, and ERK kinases in the ERK1/2 signaling pathway have been studied extensively, little is known about the activity of the ERK1/2 pathway in context of specific scaffolding modules. This dataset provides a novel look into the transcriptional response mediated through the SHOC2/ERK1/2 signaling axis, which can give greater insight into the mechanisms regulating signals of the ERK1/2 pathway [1].
• SHOC2 depletion appears to attenuate cell motility and adhesion which can be further analyzed with this data.
• Since SHOC2 is involved in the process of positively regulating RAS protein signal transduction, this dataset can be further examined to study downstream targets of RAS.
• As of 2/25/2016, only six series (including this dataset) exist in GEO with transcriptional profiles of the Cos1 cell line. This dataset becomes only the third high throughput sequencing transcriptional profile for Cos1, yielding to the potential for generalized transcriptome studies of the Cos1 cell line.

## 1 Data

This data consists of six high-throughput sequencing samples of Shoc2 depleted (*n*=3) or not depleted (*n*=3) Cos1 cells generated from an Illumina HiSeq 2000. Data is available in the Gene Expression Omnibus (GEO) [2], [3] accession GEO: GSE637063 through the direct link http://www.ncbi.nlm.nih.gov/geo/query/acc.cgi?acc=GSE67063 as well as through NCBI׳s Sequence Read Archive [4] through the direct link http://www.ncbi.nlm.nih.gov/sra?term=SRP056324.

## 2 Experimental design, materials and methods

### 2.1 Experimental design

All procedures were performed in accordance with published NIH Guidelines and the University of Kentucky Institutional Biosafety requirements. This data was designed to measure the transcriptional effects of the depletion of the SHOC2 protein within Cos1 cell lines. Control and treated cells were prepared as detailed in Section 2.2. A total of six samples were examined, with three control replicates, and three SHOC2-depleted replicates (Table 1).

### 2.2 Sample preparation

Cos1 kidney cells (American Type Culture Collection (ATCC), Manassas, VA) derived from the African green monkey (*Cercopithecidae Chlorocebus sp.)* were transduced with lentiviruses that carry non-targeting shRNA (NT) or lentiviruses carrying the shRNA targeting SHOC2 (LV1). The stable cells (Cos-NT and Cos-LV1) were grown in Dulbecco׳s Modified Eagle Medium (DMEM) with 10% Fetal Bovine Serum (FBS) supplemented with sodium pyruvate, MEM-NEAA, penicillin, streptomycin, and L-glutamate (Thermo Fisher Scientific, Waltham, MA) at 37 °C, 5% CO_2_. Cells were serum-starved for 14 h, and then treated with 0.2 ng/ml of epidermal growth factor (EGF) (BD Biosciences, San Jose, CA) for 90 min. Total RNA was extracted using Bio-Rad PureZOL/Aurum total RNA isolation kits (Bio-Rad, Hercules, CA) according to the manufacturer׳s instructions. The RNA quality was examined using an Agilent 2100 Bioanalyzer (Agilent Technologies, Santa Clara, CA). RNA-Seq libraries were constructed in the University of Texas Southwestern Genomics Core using Illumina׳s mRNA-Seq sample preparation kits (Illumina Inc., San Diego, CA) for poly-A enrichment in order to generate full mRNA sequence from any poly-A tailed RNA. The process for poly-A enrichment involved extraction of mRNA using oligo (dT) magnetic beads followed by shearing into short fragments approximately 200 bases in length. The UT Southwestern Genomics Core was responsible for mRNA isolation, cDNA synthesis, fragmentation, adaptor ligation, size selection, amplification, and quality control (QC) of the prepared libraries.

### 2.3 Data acquisition

Sequencing was performed at the University of Texas Southwestern Medical Center׳s Genomics Core using an Illumina HiSeq 2500 instrument resulting in 50 bp single end reads for each sample. Six raw sequencing files representing two conditions (control: NT and treatment: LV1) were obtained from the Illumina HiSeq 2500 instrument using the Illumina Casava basecalling software. Quality control (QC) of the raw sequence data was performed using FastQC (version 0.10.1) [5]. Based upon the QC results, minor sequence trimming was performed using Trimmomatic (version 0.27) [6] with a sliding window, trimming once the average quality within a 3-base window falls below a quality score of 20. Following trimming, QC was once again tested against the trimmed sequences. The trimmed sequences were determined to pass the QC step.

Trimmed reads were aligned to the vervet (green) monkey reference genome (*Chlorocebus sabaeus*) ChiSab1.0 (GenBank [7] accession GCA_000409795.1) downloaded from the Ensembl pre-release site (http://pre.ensembl.org/Chlorocebus_sabaeus/Info/Index) using Tophat2 v2.0.10 [8] with the multithreading option -p 4 and the remaining parameters as the default allowing for two mismatches. Tophat2 was using bowtie2 v2.1.0.0 [9] as the underlying mapper. A gene transfer format (GTF) file for the vervet monkey downloaded from the Ensembl ftp site (ftp://ftp.ensembl.org/pub/pre/gtf/chlorocebus_sabaeus/) was used as a guide for intron/exon splice junction mapping. The GTF file Chlorocebus.sabaeus.Chlsabe.0.pre.gtf was modified slightly to include “chr” within the chromosome label. Note the final GTF file contains separate genes according to whether they are annotated according to human protein homologs or *C. sabaeus* EST sequences.

Aligned RNA-seq reads were assembled onto the GTF annotation file using cufflinks (version 2.1.1) [10], resulting in a total of 51,520 genes. For each comparison, both cufflinks assemblies were merged using cuffmerge [10] and the resulting merged GTF file serves as the transcript input for differential gene expression. The number of aligned reads ranges from 82.7% to 84.3% of the original reads, indicating a high success rate (Table 2).

Differentially expressed genes were identified by comparing the combined alignments of samples 4, 5 and 6 (LV1) to the combined alignments of samples 1, 2 and 3 (NT) using cuffdiff2 (version 2.1.1) [11] with the multithreading option -p 8 and the minimum alignment count of 7 (--min-alignment-count 7) to determine gene expression levels in Fragments Per Kilobase of transcript per Megabase (FPKM) and differential expression between the two conditions. All other parameters were set to the defaults. A false-discovery rate (FDR) corrected *q*-value cutoff of 0.05 was used to determine differentially expressed genes. A list of commands used in the RNASeq pipeline is given in Table 3.

For each human gene, the corresponding Ensembl Protein ID, Gene Name, and EntrezGene ID were identified from BioMart [12] in Ensembl [13] (Ensembl Genes v77; Homo sapiens genes GrCh38). This resulting dataset was further filtered to obtain a total of 113,308 entries having values for all three fields. This data file was then used to obtain homologs to the resulting *C. sabaeus* dataset.

The resulting cuffdiff gene_exp.diff file results in 27,265 transcript identifiers from 23,709 unique regions. Note that most of the genes removed from the cufflinks portion to cuffdiff are short EST sequences. This file was parsed for differentially expressed genes defined by a *q*-value cutoff of 0.05. A total of 3907 of the genes were determined to be differentially expressed. Adding a fold-change cutoff of ±1.2 reduces this to 1987 DEGs, and a fold-change cutoff of ±1.5 reduces the list to 879 DEGs. Most of these had a human Ensembl protein homolog (3143) while some were only identified by *C. sabaeus* ESTs (764) (Table 4). A list of the top 20 differentially expressed genes is shown in Table 5.

### 2.4 Transcription factor analysis

Those genes with a human Ensembl protein homolog were further examined to identify transcription factors by cross-referencing Transfac [14] and TcoF-DB [15] databases. Transfac consists of 2301 human transcription factors. Of these, 57 are downregulated in this data set (Table 6) while 60 are upregulated (Table 7). TcoF-DB consists of transcription co-factors. The list of transcription factors and transcription co-factors were downloaded from TcoF dated 20100927. TcoF lists a total of 1365 transcription factors and 529 transcription cofactors. A total of 54 transcription co-factors were found to be differentially expressed, with 22 down-regulated (Table 8) and 32 up-regulated (Table 9).

### 2.5 Categorical enrichment

Human Entrez gene identifiers for protein homologs were used as input into categoryCompare [16] for analysis of enriched annotations including Gene Ontology [17] Biological Process (GO:BP), Molecular Function (GO:MF) and KEGG Pathways [18]. A total of 169 GO:BPs were enriched by down-regulated genes, and 188 GO:BPs were enriched by up-regulated genes. Enriched GO:MFs included 26 enriched by down-regulation, and 21 by up-regulation. In terms of KEGG Pathways, 0 were enriched for by down-regulated genes, and four were enriched by up-regulated genes. The graphical results of categoryCompare are shown in Supplementary Fig. 1 (GO:BP) and Supplementary Fig. 2 (GO:MF). Additional categorical enrichment analysis was performed by Panther [19] for GO:MF (Table 10), Gene Ontology Cellular Component (GO:CC) (Table 11), Panther Protein classes (Table 12), and Gene Ontology Biological Process (GO:BP) (Table 13). The Panther GO:MF results indicate an overall enrichment in binding and catalytic activity while the top four enriched GO:CC are cell part, organelle, membrane, and extracellular region.

## Figures and Tables

**Table 1:** Sample information.

Sample number	Sample name	Sample description	GEO sample ID
1	Cos-NT1	Non-targeting, replicate 1	GSM1637966
2	Cos-NT2	Non-targeting, replicate 2	GSM1637967
3	Cos-NT3	Non-targeting, replicate 3	GSM1637968
4	Cos-LV1	SHOC2-depleted, replicate 1	GSM1637969
5	Cos-LV2	SHOC2-depleted, replicate 2	GSM1637970
6	Cos-LV3	SHOC2-depleted, replicate 3	GSM1637971

**Table 2:** Read trimming and alignment information.

Sample name	Raw reads	Raw bases	Reads after trimming	Bases after trimming	Aligned reads	% Raw reads aligned (%)
Cos-NT1	42,952,132	2,154,871,134	38,607,172	1,501,729,353	36,207,885	84.3
Cos-NT2	38,735,131	1,942,967,564	34,782,083	1,355,688,866	32,331,306	83.5
Cos-NT3	44,044,083	2,209,603,724	39,381,284	1,528,332,458	36,772,731	83.5
Cos-LV1	43,474,834	2,180,981,048	39,011,336	1,518,169,588	36,519,959	84.0
Cos-LV2	48,201,304	2,417,879,938	43,104,467	1,675,700,194	39,878,182	82.7
Cos-LV3	40,265,842	2,019,730,082	35,969,848	1,396,805,479	33,394,305	82.9

**Table 3:** RNA-Seq pipeline commands.

Task	Command
QA/QC	fastqc <fastqFN> -o <FASTQC_DIRECTORY>
Trimming	java –classpath Trimmomatic-0.27.far TrimmomaticSE /
–phred33 <fastqIN_FN> <fastqOUT_FN> SLIDINGWINDOW:3:20
Alignment	tophat2 -p 4 -o <outputdir> -G genes.gtf /
--no-coverage-search ChSa <trimmed_fastq_file>
Transcript detection	cufflinks -p 4 -o <CUFF_OUT_FN> -G genes.gtf <CONDITION_DIR>/accepted_hits.bam
Transcript merging	cuffmerge –o cuffmerg_out –s cs1.fa samples.gtf.txt
Differential expression	cuffdiff -o cuffdiff_out -p 8 --min-alignment-count 7/
-u cuffmerg_out_gtf/merged.gtf /
Cos-NT.1_1.fastq.trim.tophat2.newFasta/accepted_hits.bam,/
Cos-NT#2_1.fastq.trim.NewFasta.tophat2/accepted_hits.bam,/
Cos-NT.3_1.fastq.trim.tophat2.newFasta/accepted_hits.bam/
Cos-LV1#4_1.fastq.trim.NewFasta.tophat2/accepted_hits.bam,/
Cos-LV1.5_1.fastq.trim.tophat2.newFasta/accepted_hits.bam,/
Cos-LV1.6_1.fastq.trim.tophat2.newFasta/accepted_hits.bam

**Table 4:** Differentially expressed genes (DEGs) as determined by cuffdiff, NT (control) vs. LV1 (SHOC2 depleted) (*q*-value ≤ 0.05).

Method	Up-regulated	Down-regulated	Total
Genes with Ensembl ID	1443	1700	3143
Unique Ensembl IDs	1367	1678	3045
*C. sabaeus* ESTs	386	378	764

**Table 5:** Top 20 differentially expressed genes (out of 853) (fold change >1.5, FDR <0.05).

**Symbol**	**Log**^**2**^**CPM**	**FPKM**	**Log ratio**	***p*-Value**	**FDR**
**AMOT**	10.37994	11758	0.683	7.23E−61	3.08E−58
**GLA**	8.609800	5982	−1.307	5.02E−153	6.15E−150
**LGALS3BP**	8.017922	4571	−1.613	8.44E−184	4.13E−180
**MYC**	7.994633	4089	−1.234	1.21E−153	1.69E−150
**NCAPG2**	8.166777	2593	0.590	1.78E−47	4.59E−45
**NEFM**	7.523503	2574	−0.683	3.22E−54	1.21E−51
**NDRG1**	7.313191	2566	−1.487	6.95E−179	2.27E−175
**SLIT2**	8.260966	2254	1.096	4.64E−171	1.14E−167
**SLC39A10**	7.265485	2199	−1.038	2.58E−31	2.87E−29
**MLLT4**	7.894645	2118	0.591	1.82E−33	2.35E−31
**NPTX1**	7.196138	2049	−0.807	1.50E−35	2.20E−33
**KRT8**	5.567323	1816	−0.880	6.29E−51	1.99E−48
**TRIB1**	6.685121	1736	−1.387	8.78E−162	1.43E−158
**TMEM164**	6.816386	1661	−0.813	1.84E−63	9.46E−61
**SORD**	6.845924	1637	−0.759	2.26E-49	6.71E−47
**CLU**	6.777606	1627	−0.779	1.41E−47	3.73E−45
**ADCY3**	6.811043	1562	−0.720	2.13E−40	4.08E−38
**CXCR4**	6.573526	1514	−1.437	1.12E−109	1.10E−106
**ALDH1A2**	6.423321	1487	−2.044	1.57E−238	1.54E−234
**TLE4**	7.369268	1437	0.714	1.21E−55	4.75E−53

**Table 6:** Down-regulated genes (*n*=57) cross-listed as transcription factors in Transfac.

AHCTF1	GTF2A1L	MAF	NFIX	SALL4	TFEB
AR	GTF2E2	MAFA	NKX1-2	SOX5	TP53
CAV1	GTF2F2	MAMSTR	NKX6-1	SP1	TRIM21
CDH1	HES5	MEF2C	NR2F2	SP4	UBE3A
DACH2	HES7	MITF	OLIG2	SREBF1	XBP1
E2F4	HIVEP3	MLX	OVOL2	STAT5B	ZBTB7B
E2F5	JUNB	MYB	PITX3	TBX1	ZNF143
ELF4	KLF6	MYC	POU3F2	TCEAL3	
FOXN2	KLF9	MYPOP	POU3F3	TCF24	
GHR	LSR	NFATC2	PRR5	TFB2M	

**Table 7:** Up-regulated genes (*n*=60) cross-listed as transcription factors in Transfac.

ACE2	DMRT3	FOXP2	LBX1	PBRM1	TCEANC2
ATF5	EBF2	GTF2A1	LMX1B	PBXIP1	TCF7L2
BARD1	ELP2	GTF2H5	LZTFL1	PIR	TFAP2A
BCLAF1	EPAS1	GTF2I	MTF1	POU2F1	TP73
CDX2	ETV3	GTF3C3	NFAT5	PPARD	TRRAP
CNOT4	FHL1	HINFP	NFKBIZ	PRDM16	TWIST2
CNOT6	FLI1	IFI16	NFYA	SALL2	VIM
CREBBP	FOXC1	ISL1	NFYC	SPEN	XRCC4
CTBP1	FOXF2	KLF12	NR2F1	SUPT16H	YAP1
DACH1	FOXO4	L3MBTL1	NR6A1	TAX1BP3	ZEB1

**Table 8:** Down-regulated genes (*n*=22) cross-listed as transcription co-factors in TcoF.

AGO2	EYA2	HMGA2	NAB2	SAP30	THAP1
BCL3	FHL2	MAML3	NUP62	SIAH2	TRIB3
EID2	HDAC8	MBD2	PTRF	SSBP2	
ERBB4	HDAC9	MTA3	NAB2	TGFB1	

**Table 9:** Up-regulated genes (*n*=32) cross-listed as transcription co-factors in TcoF.

ATN1	CRY1	HIPK2	PEX14	POGZ	SSBP3	TXNIP
BRD8	CTBP1	KDM5A	PHF1	RING1	STK36	YAP1
CBX8	ELP2	MED20	PIR	SMAD6	TAF9B	
CHD4	ERCC6	MED21	PNRC1	SMAD7	TLE4	
CHD8	HCFC1	NSD1	PNRC2	SNIP1	TRRAP	

**Table 10:** Gene Ontology Molecular Function (GO:MF) enriched categories determined by Panther.

**GO Molecular Function**	**# of genes**	**Percentage (%)**
Binding (GO:0005488)	272	32.60
Catalytic activity (GO:0003824)	211	25.30
Nucleic acid binding transcription factor activity (GO:0001071)	102	12.20
Receptor activity (GO:0004872)	89	10.70
Transporter activity (GO:0005215)	62	7.40
Structural molecule activity (GO:0005198)	45	5.40
Enzyme regulator activity (GO:0030234)	42	5.00
Protein binding transcription factor activity (GO:0000988)	8	1.00
Translation regulator activity (GO:0045182)	1	0.10
Channel regulator activity (GO:0016247)	1	0.10
Antioxidant activity (GO:0016209)	1	0.10

**Table 11:** Gene Ontology Cellular Component (GO:CC) enriched categories determined by Panther.

**GO Cellular Component**	**# of genes**	**Percentage (%)**
Cell part (GO:0044464)	130	36.00
Organelle (GO:0043226)	74	20.50
Membrane (GO:0016020)	53	14.70
Extracellular region (GO:0005576)	48	13.30
Extracellular matrix (GO:0031012)	26	7.20
Macromolecular complex (GO:0032991)	22	6.10
Synapse (GO:0045202)	4	1.10
Cell junction (GO:0030054)	4	1.10

**Table 12:** Panther Protein Class enriched categories.

**Panther Protein Class**	**# of Genes**	**Percentage (%)**
Nucleic acid binding (PC00171)	102	11.30
Transcription factor (PC00218)	98	10.80
Receptor (PC00197)	92	10.20
Hydrolase (PC00121)	72	7.90
Transferase (PC00220)	67	7.40
Signaling molecule (PC00207)	63	7.00
Transporter (PC00227)	53	5.80
Enzyme modulator (PC00095)	53	5.80
Cytoskeletal protein (PC00085)	34	3.80
Oxidoreductase (PC00176)	34	3.80
Kinase (PC00137)	29	3.20
Protease (PC00190)	28	3.10
Extracellular matrix protein (PC00102)	27	3.00
Cell adhesion molecule (PC00069)	22	2.40
Defense/immunity protein (PC00090)	19	2.10
Transfer/carrier protein (PC00219)	18	2.00
Membrane traffic protein (PC00150)	18	2.00
Calcium-binding protein (PC00060)	17	1.90
Phosphatase (PC00181)	11	1.20
Ligase (PC00142)	10	1.10
Structural protein (PC00211)	10	1.10
Cell junction protein (PC00070)	8	0.90
Transmembrane receptor regulatory/adaptor protein (PC00226)	6	0.70
Lyase (PC00144)	6	0.70
Surfactant (PC00212)	4	0.40
Chaperone (PC00072)	3	0.30
Storage protein (PC00210)	1	0.10
Isomerase (PC00135)	1	0.10

**Table 13:** Gene Ontology Biological Process (GO:BP) enriched categories determined by Panther.

**Panther Biological Process**	**# of genes**	**Percentage (%)**
Metabolic process (GO:0008152)	351	22.00
Cellular process (GO:0009987)	324	20.30
Biological regulation (GO:0065007)	210	13.10
Developmental process (GO:0032502)	166	10.40
Localization (GO:0051179)	125	7.80
Multicellular organismal process (GO:0032501)	109	6.80
Response to stimulus (GO:0050896)	93	5.80
Immune system process (GO:0002376)	75	4.70
Cellular component organization or biogenesis (GO:0071840)	49	3.10
Biological adhesion (GO:0022610)	38	2.40
Apoptotic process (GO:0006915)	30	1.90
Reproduction (GO:0000003)	23	1.40
Locomotion (GO:0040011)	5	0.30
Growth (GO:0040007)	1	0.10

## Appendix A Supplementary material

Supplementary material

GO:BP enrichments for differentially expressed genes, as determined by categoryCompare.

GO:MF enrichments for differentially expressed genes, as determined by categoryCompare.

## Footnotes

Appendix A Supplementary data associated with this article can be found in the online version at doi:10.1016/j.dib.2016.03.012.
