# Supplementary figures and images for: Data set for transcriptional response to depletion of the Shoc2 scaffolding protein
Source: Data Brief. 2016 Mar 9;7:770–8. doi: 10.1016/j.dib.2016.03.012 (PMC4816878; doi:10.1016/j.dib.2016.03.012)

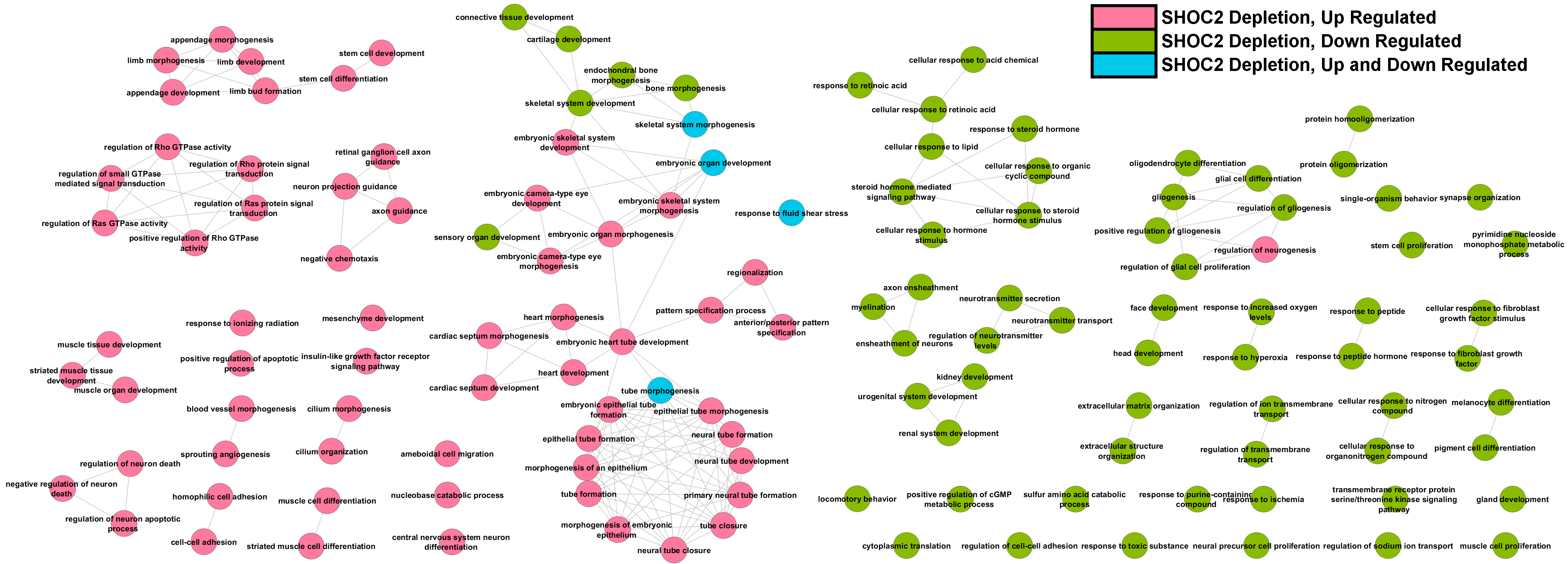

Supplement: Supplementary file 2 — GO:BP enrichments for differentially expressed genes, as determined by categoryCompare. [file mmc2.zip › Figure1-GOBP_SHOC2_EMILIAwithKEY.png]

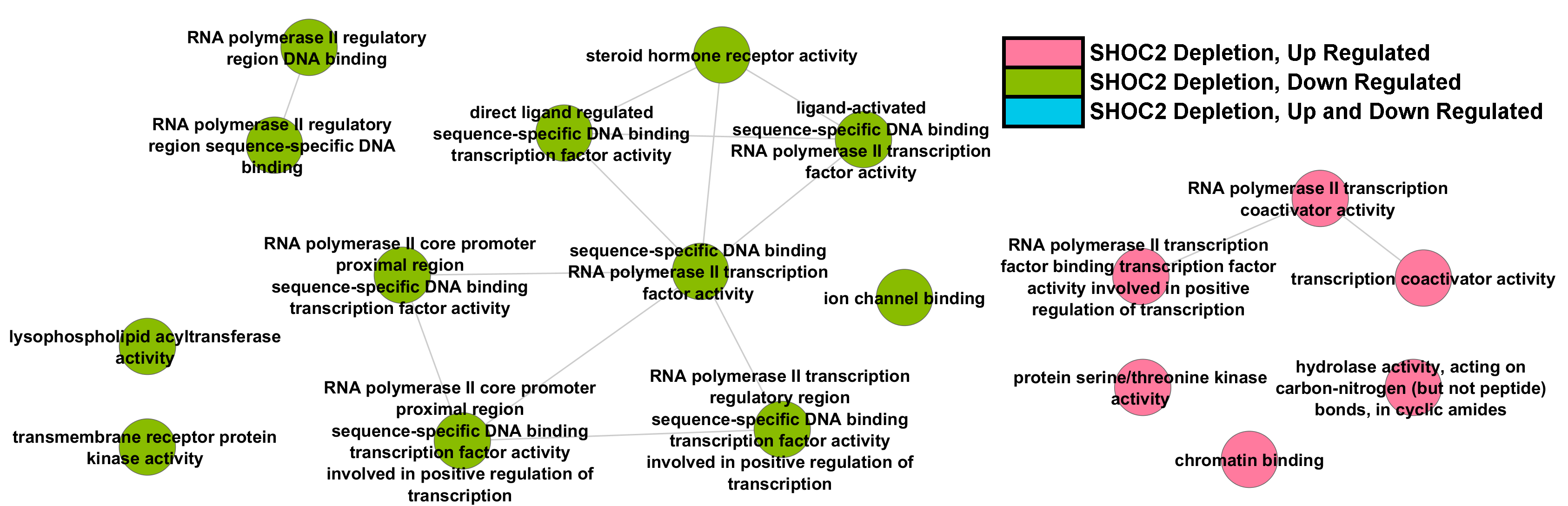

Supplement: Supplementary file 3 — GO:MF enrichments for differentially expressed genes, as determined by categoryCompare. [file mmc3.zip › Figure2-GOMF_SHOC2_EMILIAwithKEY.png]
